# The hidden path of mycobacterium: a case series of rare and elusive manifestations of gastrointestinal tuberculosis

**DOI:** 10.17179/excli2025-9040

**Published:** 2026-01-05

**Authors:** Nitika Yadav, Abhishek Yadav, Neharica Joshi, Shubhangi Gupta, Yashendra Sethi

**Affiliations:** 1 Subharti Medical College, Swami Vivekanand Subharti University, Meerut, India; 2 PearResearch, Dehradun, India; 3 Lumen Foundation, Florida, US

**Keywords:** anti-tuberculosis treatment, tuberculosis, GI TB, duodenal TB, abdominal TB

## Abstract

Extrapulmonary tuberculosis continues to challenge clinicians with its protean manifestations, particularly when involving the gastrointestinal tract. While ileocecal TB is well-characterized, isolated involvement of the liver, stomach, and esophagus, Duodenum remains exceptionally rare and diagnostically elusive. We describe four rare presentations of gastrointestinal tuberculosis from a tertiary care center in India. Case 1 involves hepatic tuberculosis mimicking intrahepatic cholestasis, diagnosed via liver biopsy and special staining. Case 2A details isolated gastric TB presenting with nonspecific dyspepsia, ultimately diagnosed through endoscopic ultrasound-guided FNAC. Case 2B describes esophageal tuberculosis with bronchoesophageal fistula-an exceedingly rare entity-confirmed radiologically and histologically. Case 2C describes a case of duodenal tuberculosis presenting as partial gastric outlet obstruction (GOO). All cases demonstrated clinical and radiological resolution following standard anti-tubercular therapy. These cases posit the diagnostic complexity of gastrointestinal TB when it involves uncommon sites. Heightened clinical suspicion and timely histopathological confirmation remain key to averting morbidity. Early recognition facilitates successful medical management and obviates unnecessary surgical interventions.

## What is already Known

- Gastrointestinal tuberculosis (GI TB) is a rare extrapulmonary manifestation, with most cases involving the ileocecal region.
- Hepatic, gastric, and esophageal TB are exceptionally rare, comprising <1% of all abdominal TB presentations combined [WHO, 2022[12]; Choudhury et al., 2023[3]; Horvath and Whelan, 1998[5]; Anand et al., 1992[2]].
- Diagnosis is frequently delayed due to nonspecific symptoms and overlapping features with malignancy, inflammatory bowel disease, or cryptogenic infections.
- Histopathology remains the diagnostic cornerstone, with caseating granulomas and AFB positivity providing definitive evidence.

## What this Case Series Adds

- This is among the few Indian case series to document four anatomically distinct and rare GI TB forms-isolated hepatic TB, primary gastric TB, and esophageal TB with bronchoesophageal fistula and Duodenal tuberculosis.
- All three cases occurred in immunocompetent patients without active pulmonary TB, emphasizing the need to consider TB in atypical abdominal presentations even without respiratory involvement.
- Early use of targeted biopsies (including liver core biopsy and EUS-FNAC) and a low threshold for invasive diagnostics were pivotal in timely diagnosis.
- The remarkable therapeutic response to standard antitubercular therapy (ATT) in all cases, including a bronchoesophageal fistula that resolved without surgical intervention, underscores the potential for conservative management, even in anatomically complex disease.
- This series highlights key clinical and biochemical red flags (e.g., cholestatic LFTs, unexplained dysphagia, submucosal gastric nodules) that should prompt consideration of GI TB in endemic settings.

## Introduction

Tuberculosis (TB) remains a major global health burden, with an estimated 10.6 million cases reported in 2021, disproportionately affecting low- and middle-income countries (WHO, 2022[12]). While pulmonary TB constitutes the majority, extrapulmonary tuberculosis (EPTB) accounts for approximately 15-20 % of all TB cases, with gastrointestinal involvement reported in 1-3 % (Choudhury et al., 2023[3]). Within the gastrointestinal system, the ileocecal region is the most commonly affected site, owing to its abundant lymphoid tissue and prolonged transit time (Horvath and Whelan, 1998[5]).

Isolated hepatic, gastric, duodenal or esophageal involvement is rare, often overlooked due to nonspecific presentations and insidious progression. These forms pose diagnostic challenges owing to their mimicry of more common hepatobiliary, neoplastic, or inflammatory conditions. Histopathological confirmation and targeted imaging are pivotal in reaching a definitive diagnosis.

Herein, we present four clinically and pathologically distinct cases of abdominal TB: hepatic tuberculosis presenting with cholestatic jaundice and malnutrition, isolated gastric TB mimicking malignancy, esophageal TB complicated by a bronchoesophageal fistula and Duodenal tuberculosis, though exceedingly rare, mimicking as gastric outlet obstruction-posing significant diagnostic delay. Through this series, we aim to emphasize the diagnostic complexity, therapeutic responsiveness, and clinical implications of these atypical TB presentations.

## Case Presentation

### Case 1: Hepatic tuberculosis masquerading as cholestatic hepatopathy

A 21-year-old woman, resident of urban North India, presented to our tertiary care hospital with one month of low-grade, intermittent fever and mild right hypochondriac abdominal discomfort described as a dragging sensation. The illness was insidious, accompanied by profound anorexia and unintentional weight loss. Notably, there was no history of cough, urinary symptoms, jaundice, night sweats, or changes in bowel habits.

On examination, the patient was thin-built (BMI 16.8 kg/m²) and pale, without peripheral lymphadenopathy or icterus. Abdominal palpation revealed hepatomegaly without guarding or tenderness. Systemic examination was otherwise unremarkable except for signs of protein-energy malnutrition. Initial laboratory investigations (Table 1(Tab. 1)) revealed normocytic anemia (Hb 7.4 g/dL), elevated liver enzymes predominantly in a cholestatic pattern-markedly elevated alkaline phosphatase (907 U/L) and γ-glutamyl transpeptidase (542 U/L)-with mildly elevated bilirubin and AST. Over the subsequent weeks, there was a fluctuating but persistent pattern of deranged liver biochemistry. Viral serologies for hepatitis B, C, and HIV were negative. Autoimmune workup, including antinuclear antibody (ANA) testing by immunofluorescence, was also negative. Ultrasonography of the abdomen showed hepatomegaly (15 cm), a thickened gallbladder wall, mild ascites, and splenomegaly. Chest X-ray was unremarkable except for mild cardiomegaly and blunting of the right costophrenic angle. Echocardiography revealed a mild circumferential pericardial effusion and mild pulmonary artery hypertension.

**Figure 1:**
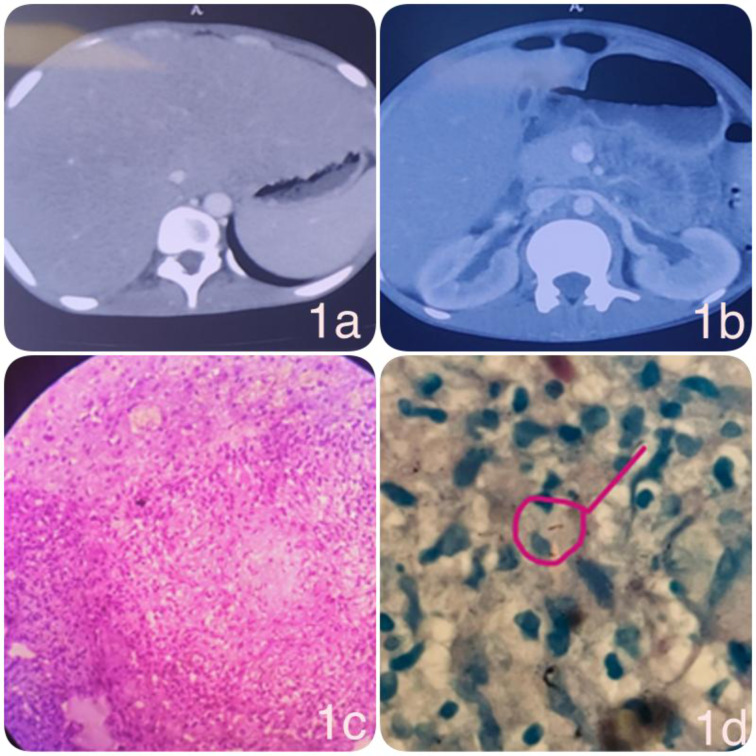
a, b) CECT abdomen images showing massive hepatomegaly with periportal lymphadenopathy. c, d) H&E section shows caseating granuloma composed of lymphocytic aggregates and AFB positivity on Zn stain.

Due to persistently worsening liver function and no clear etiology, a contrast-enhanced CT of the abdomen was performed (Figure 1a(Fig. 1)), revealing significant hepatomegaly (19 cm), periportal cuffing, and enlarged periportal lymph nodes with a thickened gallbladder wall but no biliary dilatation. In view of diagnostic uncertainty, the patient was referred to the Department of Gastroenterology for further evaluation. Following multidisciplinary discussion and informed consent, she underwent a percutaneous ultrasound-guided liver biopsy along with fine-needle aspiration cytology (FNAC) of periportal lymph nodes.

Histopathological examination of the liver biopsy (Figure 1b(Fig. 1)) revealed non-caseating and caseating granulomas composed of epithelioid cells and Langhans-type multinucleated giant cells. Special staining with Ziehl-Neelsen (ZN) confirmed the presence of acid-fast bacilli (AFB), consistent with Mycobacterium tuberculosis infection. Periodic acid-Schiff (PAS) stain and Congo red stain for amyloid were negative, ruling out fungal or amyloid-related pathologies.

The diagnosis of hepatic tuberculosis with diffuse granulomatous inflammation and intrahepatic cholestasis was established. The patient was initiated on a standard four-drug anti-tubercular therapy (ATT) regimen: isoniazid (5 mg/kg/day), rifampicin (10 mg/kg/day), pyrazinamide (25 mg/kg/day), and ethambutol (15 mg/kg/day), along with supportive nutrition therapy and pyridoxine.

Over the ensuing 4 weeks, the patient demonstrated significant clinical improvement with resolution of fever, increased appetite, and weight gain. Follow-up investigations at 4 and 8 weeks revealed progressive normalization of liver function parameters, as shown in Table 2(Tab. 2). Repeat ultrasonography at 3 months showed regression of hepatosplenomegaly and disappearance of ascites.

### Case 2A: A stomach without clues - isolated gastric tuberculosis mimicking submucosal disease

A 20-year-old woman with no significant past medical history presented to our outpatient department with complaints of dull epigastric pain persisting for two months. The pain was described as intermittent, non-radiating, and poorly localized, without clear aggravating or relieving factors. She also reported profound anorexia and unintended weight loss of approximately 6 kg during the same period.

There was no associated history of nausea, vomiting, hematemesis, melena, or altered bowel habits. Importantly, she denied any fever, respiratory symptoms, or constitutional complaints such as night sweats. There was no personal or family history of tuberculosis, and she had not received Bacillus Calmette-Guérin (BCG) vaccination in childhood. The patient was not on any medications and had no history of immunosuppression or contact with a known TB patient.

On clinical examination, she appeared pale and undernourished, with a BMI of 17.2 kg/m². Mild epigastric tenderness was noted on deep palpation, without palpable mass or organomegaly. There was no lymphadenopathy, ascites, or peripheral stigmata of chronic illness. Cardiopulmonary and neurological examinations were within normal limits.

Initial laboratory investigations revealed microcytic anemia with hemoglobin of 7.1 g/dL, while leukocyte counts and differential were within normal limits. Liver and renal function panels were unremarkable. ESR was elevated at 22 mm/h, and the Mantoux test was strongly positive at 15 mm. Viral serologies for HIV, hepatitis B, and C were negative.

Ultrasonography of the abdomen revealed thickening of the gastric wall, predominantly in the pyloric and antral regions. This finding prompted a contrast-enhanced CT (CECT) scan of the abdomen, which showed circumferential thickening of the gastric body and antrum with mild perigastric stranding and subcentimetric lymphadenopathy. These findings raised differential considerations of gastric lymphoma, infiltrative carcinoma, or granulomatous disease.

Upper gastrointestinal endoscopy was performed, revealing thickened, velvety gastric folds in the body and antrum, without ulceration or bleeding. No mass lesion or obstruction was visualized. Endoscopic biopsy was inconclusive due to superficial sampling. Subsequently, an endoscopic ultrasound (EUS) was conducted, revealing homogeneous thickening of the third layer of the gastric wall (muscularis propria) without nodal involvement or serosal breach. EUS-guided fine-needle aspiration cytology (FNAC) was performed from the thickened gastric wall.

**Figure 2:**
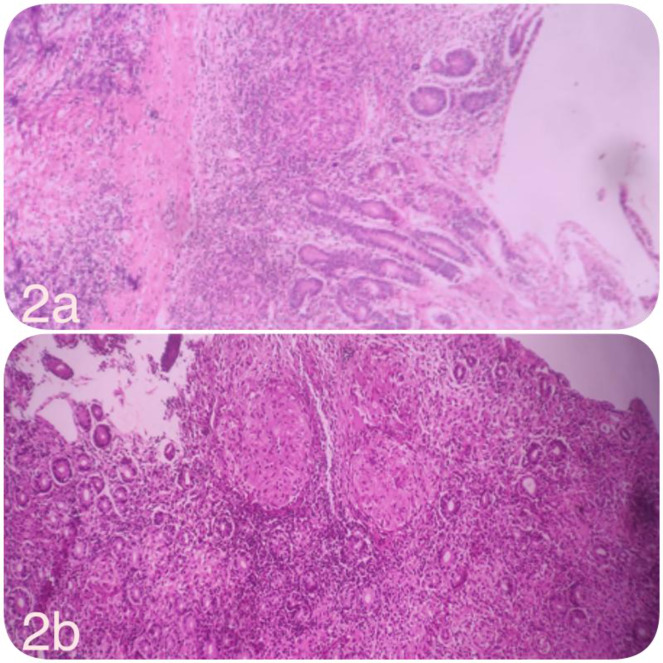
a) Hematoxylin and eosin (H&E)-stained gastric biopsy specimen demonstrating preserved gastric glands interspersed with well-formed epithelioid cell granulomas. The granulomas are non-caseating, surrounded by a sparse lymphocytic infiltrate, and localized within the lamina propria. b) H&E-stained section showing intact gastric glands and pits with a discrete submucosal epithelioid granuloma. The granuloma demonstrates compact aggregates of epithelioid histiocytes with peripheral lymphocytic cuffing, consistent with granulomatous inflammation.

Cytological analysis of the aspirate demonstrated multiple epithelioid cell granulomas with central necrosis and surrounding lymphoplasmacytic infiltrate (Figure 2a, b(Fig. 2)). Occasional Langhans-type giant cells were observed. The final diagnosis of isolated gastric tuberculosis was made.

The patient was initiated on first-line anti-tubercular therapy (isoniazid, rifampicin, pyrazinamide, and ethambutol) , along with pyridoxine and iron supplementation for anemia. Nutritional rehabilitation with high-protein diet and oral supplements was also implemented.

Over the course of 12 weeks, the patient demonstrated progressive clinical recovery. She reported complete resolution of abdominal pain and gained 10 kg in body weight. Repeat endoscopy at 3 months showed marked reduction in gastric wall thickening and normalization of the mucosal folds. Follow-up laboratory testing demonstrated a rise in hemoglobin to 11.6 g/dL, normalized ESR, and improved nutritional markers.

### Case 2B: An esophageal tuberculosis presenting with dysphagia

A 48-year-old man presented to our outpatient clinic with a primary complaint of progressive dysphagia to solid foods over the preceding two months. Initially intermittent, the dysphagia had become persistent and was associated with vague, non-radiating retrosternal and epigastric discomfort. He denied odynophagia, regurgitation, vomiting, or symptoms suggestive of gastroesophageal reflux.

No history of cough for six weeks, without hemoptysis, diurnal variation, or positional influence. The patient also reported unintended weight loss (approx. 5 kg), reduced appetite, and low-grade evening fevers. There were no prior episodes of TB, nor a history of close contact with a TB patient. He had no history of dyspepsia, abdominal pain, altered bowel habits, or systemic features like joint pain or rash. There was no history of substance abuse or long-term medication use.

Physical examination revealed a BMI of 21.5 kg/m². There was no pallor, or lymphadenopathy or clubbing. Chest auscultation was unremarkable breath sounds over the right upper lung field. Cardiovascular and abdominal examinations were unremarkable.

Initial laboratory evaluation (Table 3(Tab. 3)) revealed hemoglobin of 13.0 g/dL, total leukocyte count of 7500/mm³ with a normal differential, and ESR of 65 mm/hr. Liver and renal function tests were within normal limits. Mantoux test was positive (10 mm). Viral markers for HIV, hepatitis B, and C were negative.

In view of persistent dysphagia, an esophagogastroduodenoscopy (EGD) was performed, revealing discrete ulcerated lesions in the esophagus at 25, cm from the incisors (Figure 1(Fig. 1)). The ulcers were shallow, irregular, and surrounded by hyperemic mucosa. No strictures or mass lesions were observed. Biopsies were taken from the ulcer margins. A contrast-enhanced computed tomography (CT) scan of the chest revealed short segment circumferential thickening of the esophageal wall,. Additionally, multiple enlarged Pretracheal , subcranial regions.

**Figure 3:**
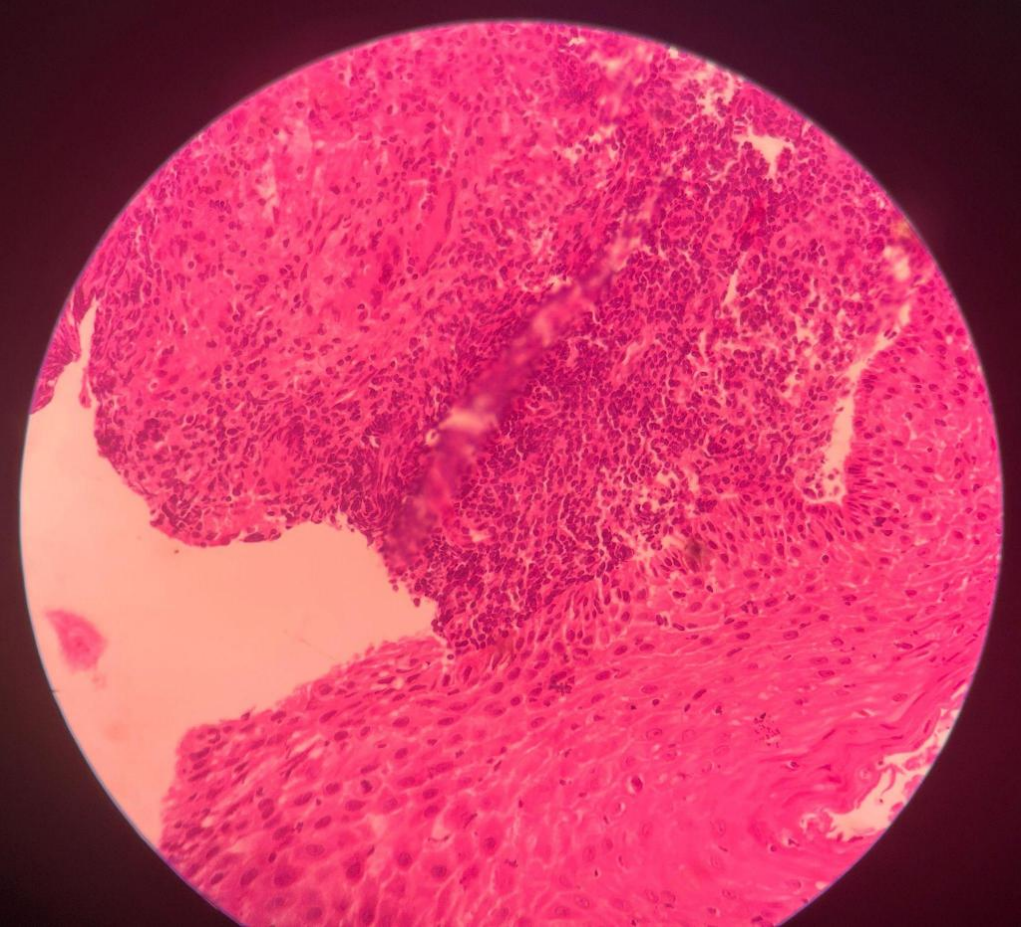
Hematoxylin and eosin (H&E)-stained section of the esophageal biopsy showing stratified squamous epithelium with an underlying granulomatous infiltrate composed of lymphocytes and epithelioid histiocytes, including multinucleated Langhans giant cells.

Histopathological analysis of esophageal biopsy (Figure 3(Fig. 3)) revealed hyperplastic squamous epithelium with focal erosions dense infiltration of inflammatory cells subepithelial granulomatous inflammation comprising epithelioid cells, Langhans giant cells, and lymphocytic infiltrates (Figure 3(Fig. 3)). Overall feature suggestive of Chronic Granulomatous esophagitis Although AFB staining was negative, the combination of granulomatous inflammation, positive Mantoux test, and suggestive radiologic features clinched the diagnosis of esophageal tuberculosis.

**Figure 4:**
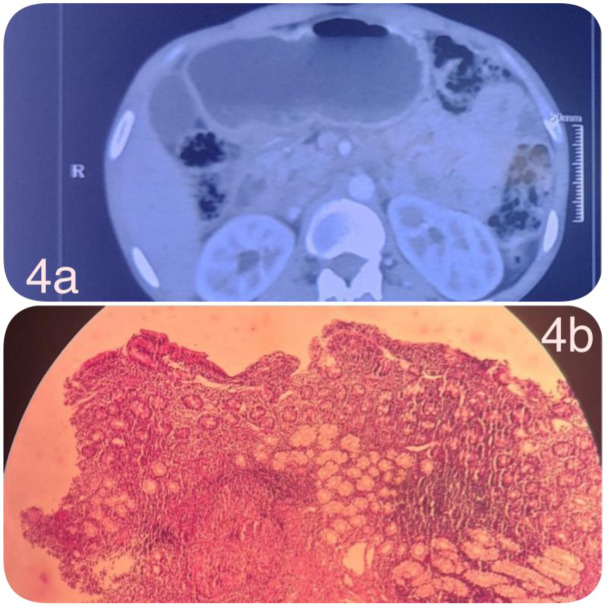
a) Contrast-enhanced computed tomography (CECT) of the abdomen demonstrating concentric mural thickening of the proximal duodenum, associated with multiple enlarged periduodenal and mesenteric lymph nodes showing central necrosis, a radiologic feature highly suggestive of abdominal tuberculosis. b) Hematoxylin and eosin (H&E)-stained section from a duodenal biopsy revealing Brunner's glands with a well-formed submucosal epithelioid granuloma, consistent with granulomatous duodenitis.

The patient was initiated on a four-drug anti-tubercular therapy (isoniazid 5 mg/kg, rifampicin 10 mg/kg, pyrazinamide 25 mg/kg, and ethambutol 15 mg/kg daily), along with pyridoxine. Nutritional support and cough suppression were also instituted. Clinical improvement was observed within four weeks, with resolution of fever and weight stabilization. At the 8-week mark, a repeat EGD demonstrated near-complete mucosal healing with resolution of esophageal ulcers (Figure 4(Fig. 4)). A follow-up CT chest confirmed radiologic clearance of pulmonary lesions. Laboratory markers also normalized (Table 4(Tab. 4)), and the patient remained asymptomatic on ATT.

### Case 2C: Granulomatous duodenitis causing partial gastric outlet obstruction in a young male

A 24-year-old male from western Uttar Pradesh presented with persistent non-bilious vomiting for over one month, associated with dull upper abdominal pain and early satiety. He reported progressive postprandial fullness and weight loss of nearly 5 kg over two months. There was no history of fever, hematemesis, melena, dysphagia, odynophagia, altered bowel habits, alcohol use, NSAID intake, or prior anti-tubercular therapy. He denied any history of chronic cough, contact with a tuberculosis case, or prior gastrointestinal illness. He had sought treatment from a local practitioner and was prescribed a proton-pump inhibitor and anti-emetics, with no symptomatic improvement. On presentation, he appeared mildly dehydrated but was afebrile, with stable vital signs. Abdominal examination revealed a soft but distended epigastrium with visible succussion splash; there was no palpable mass or organomegaly. Digital rectal examination was unremarkable.

Laboratory investigations revealed microcytic anemia (hemoglobin 9.1 g/dL, MCV 78 fL), normal total leukocyte and platelet counts, and preserved liver and renal function. Serum amylase, lipase, calcium, and electrolytes were within normal range. HIV, HBsAg, and anti-HCV serologies were negative. Erythrocyte sedimentation rate (ESR) was mildly elevated (38 mm/hr), while C-reactive protein was normal.

An upper gastrointestinal endoscopy revealed circumferential mucosal edema and thickening at the D1-D2 junction, causing significant luminal narrowing and stasis of gastric contents suggestive of partial gastric outlet obstruction. No ulcer or mass lesion was seen. Multiple biopsies were taken from the affected area.Contrast-enhanced CT of the abdomen revealed concentric mural thickening of the proximal duodenum, with multiple enlarged periduodenal and mesenteric lymph nodes exhibiting central necrosis. There was no pulmonary lesion or abdominal ascites. Histopathological examination showed chronic granulomatous inflammation with caseous necrosis, consistent with tuberculosis. Ziehl-Neelsen staining revealed occasional acid-fast bacilli. No dysplasia or malignancy was noted (Figure 4a, b(Fig. 4)).

The patient was diagnosed with isolated duodenal tuberculosis causing partial gastric outlet obstruction. He was initiated on standard weight-based anti-tubercular therapy (isoniazid, rifampicin, pyrazinamide, and ethambutol). Nutritional support was provided with a liquid diet. Over the next six weeks, his symptoms improved significantly, and he regained 2.5 kg. A follow-up endoscopy at 8 weeks showed partial resolution of the duodenal narrowing, and therapy was continued for six months with complete clinical resolution. Under 30 cases have been reported on Duodenal tuberculosis and this forms an extremely rare entity. Other cases reviewed are compiled in Supplementary table 1.

## Discussion

Tuberculosis (TB), a global public health challenge, continues to defy diagnostic norms through its extrapulmonary and atypical manifestations. Gastrointestinal tuberculosis (GI TB) accounts for up to 3 % of all TB cases, yet isolated involvement of the liver, stomach, or esophagus remains exceedingly rare, often mimicking neoplastic, autoimmune, or cryptogenic disorders [1-3]. The cases in this series-isolated hepatic TB, gastric TB mimicking a submucosal mass, and esophageal TB -highlight the protean nature of TB and the central role of tissue diagnosis and clinical suspicion in endemic settings.

**The first case**, a patient with isolated hepatic TB, presented with prolonged fever, anorexia, and hepatomegaly without pulmonary symptoms. While hepatic TB can manifest in miliary, nodular, or diffuse parenchymal forms, isolated hepatic involvement without concurrent pulmonary or miliary disease is distinctly uncommon (Alvarez, 1998[1]). The clinical picture of disproportionate alkaline phosphatase elevation with relatively preserved aminotransferases suggested a cholestatic or infiltrative process. In such settings, liver biopsy is diagnostic, revealing caseating granulomas with or without AFB positivity. This case reinforces the need to pursue early histological evaluation in patients with undiagnosed pyrexia and hepatic dysfunction in endemic areas, even in the absence of typical TB findings on imaging (Essop et al., 1984[4]; Tai et al., 2005[11]).

**The second case** involved primary gastric tuberculosis, one of the rarest forms of GI TB with an incidence of <0.2 % even in endemic zones (Zhang et al., 2022[13]). The stomach's acidic environment, relative lymphoid paucity, and continuous epithelial turnover are traditionally considered inhospitable to Mycobacterium tuberculosis. Gastric TB typically occurs via secondary spread from pulmonary or lymph nodal disease, but our patient had no such primary focus. CT imaging suggested a submucosal mass, raising suspicion for malignancy or lymphoma. However, endoscopic ultrasound-guided FNAC revealed necrotizing granulomatous inflammation with AFB positivity, confirming TB. This case exemplifies the diagnostic value of EUS-guided sampling in distinguishing deep mural TB from neoplasms and expands the differential diagnosis of gastric masses in TB-endemic areas (Lv et al., 2020[6]; Sharma and Bhatia, 2004[9]; Anand et al., 1992[2]).

**The third case** described esophageal TB, a condition comprising less than 0.3 % of GI TB presentations (Mokoena et al., 1992[7]). Most commonly resulting from direct extension from mediastinal lymph nodes, esophageal TB typically involves the middle third of the esophagus and may present with dysphagia, odynophagia, retrosternal pain, or systemic symptoms (Singh et al., 2017[10]). The diagnosis was established through a combination of CT, upper GI endoscopy, and biopsy-despite negative AFB staining. Conservative management with standard anti-tubercular therapy alone resulted in complete resolution of systemic symptoms, obviating the need for surgical intervention. This supports emerging literature that BEF secondary to TB, if detected early, may respond well to medical therapy alone (Choudhury et al., 2023[3]).

**Fourth case** -Duodenal tuberculosis is one of the rarest forms of gastrointestinal TB, typically involving the first and second portions due to regional lymphatic drainage. It can present with vomiting, early satiety, and weight loss due to partial or complete gastric outlet obstruction, mimicking pyloric malignancy or complicated peptic disease. Radiologic findings may include circumferential thickening of the duodenum and necrotic lymphadenopathy, but diagnosis often relies on endoscopic biopsy. Caseating granulomas with AFB positivity confirm diagnosis. Our case highlights that timely biopsy and early ATT initiation can lead to resolution, avoiding surgical intervention (Sato et al., 2024[8]).

Together, these cases underscore the following clinical principles. First, tuberculosis must remain high on the differential diagnosis of any unexplained hepatogastrointestinal pathology in endemic settings, particularly when symptoms persist despite standard therapy. Second, minimally invasive diagnostic tools-such as image-guided FNAC or EUS-biopsy-are indispensable in evaluating suspected deep-seated lesions. Third, microbiological confirmation, while ideal, is not always feasible; characteristic histopathology (necrotizing granulomas) remains highly specific in the right clinical context (Essop et al., 1984[4]; Tai et al. 2005[11]; Lv et al., 2020[6]). Finally, early initiation of anti-tubercular therapy can yield excellent outcomes, even in anatomically complex cases. Cases show amazing response to ATT even in presence of anatomical symptoms like case 2c and cases listed in Supplementary table 1.

A consolidated overview of atypical gastrointestinal TB presentations, including their clinical, radiological, and histopathological features, along with diagnostic pitfalls and management pearls, is provided in Table 5(Tab. 5). Table 6(Tab. 6) outlines a practical framework for recognizing and managing atypical gastrointestinal TB, emphasizing red-flag symptoms and high-yield diagnostic pathways tailored for endemic settings. Table 7(Tab. 7) highlights the spectrum of histopathological findings in abdominal tuberculosis and their mimics, emphasizing the importance of clinicopathologic correlation and appropriate staining techniques in achieving diagnostic certainty

These cases reflect not just the diversity of tuberculosis but its enduring relevance as a clinical masquerader. In a post-pandemic world where diagnostic inertia and empiricism threaten to eclipse detailed evaluation, the clinician must remain vigilant for TB's subtler manifestations-often hiding in plain sight.

### Clinical practice points

- Always consider hepatic TB in patients from endemic regions presenting with fever, hepatomegaly, and a cholestatic liver profile, especially if initial imaging is non-diagnostic.
- Gastric TB can mimic malignancy; endoscopic ultrasound-guided FNAC increases diagnostic yield in submucosal or nodular lesions.
- Negative AFB staining does not exclude GI TB; histopathologic features like caseating granulomas are often sufficient for diagnosis.
- Early invasive diagnostics (biopsy, EUS) and empirical ATT in high-suspicion cases are key in avoiding complications and surgery.

### Public health implications

These cases underscore the continued burden of extrapulmonary TB even in immunocompetent adults without pulmonary disease. Delayed diagnosis due to nonspecific symptoms and diagnostic inertia can lead to complications requiring invasive interventions. Our findings support the integration of early tissue diagnosis-particularly liver biopsy and EUS-guided sampling-into diagnostic pathways in endemic settings. In resource-limited regions, clinical suspicion combined with empirical ATT may be warranted when histology is suggestive, even if microbiologic confirmation is lacking.

### Mechanistic insight: why are these sites so rarely involved?

- Liver: High vascularity and reticuloendothelial function make it a filtration site, yet primary involvement is rare due to robust immune surveillance.
- Stomach: Acidic environment, fast mucosal turnover, and limited lymphoid tissue limit TB colonization.
- Esophagus: Stratified squamous epithelium, lack of mucosal lymphoid aggregates, and peristalsis offer mechanical and immunologic resistance.
- When TB does occur, it's often due to lymphohematogenous spread or direct extension from adjacent structures, especially in high-inoculum exposure or compromised mucosal defenses.

## Conclusion

This case series highlights the protean and often deceptive nature of abdominal tuberculosis, particularly in its rare manifestations involving the liver, stomach, duodenum and esophagus. These atypical presentations, though infrequent, carry significant diagnostic delays due to their mimicry of neoplasia, lack of classical features, and the limitations of standard non-invasive tests. In all four cases, definitive diagnosis hinged on timely tissue acquisition-whether by liver biopsy, EUS-guided FNAC, or endoscopic sampling-and characteristic histopathological findings, even in the absence of microbiological confirmation. Favorable outcomes with standard antitubercular therapy further affirm the importance of maintaining a high index of suspicion in endemic regions. The reversibility of gastric outlet obstruction with medical therapy alone in duodenal TB emphasizes the importance of early recognition and biopsy in patients with unexplained proximal intestinal obstruction in endemic regions. Clinicians should remain vigilant for extrapulmonary TB in patients presenting with unexplained gastrointestinal or hepatobiliary symptoms, as early diagnosis can obviate surgical intervention and improve outcomes. Our findings also underscore the need for updated diagnostic algorithms, education on uncommon presentations, and a nuanced approach to empirical therapy in resource-constrained settings.

**Figure 5:**
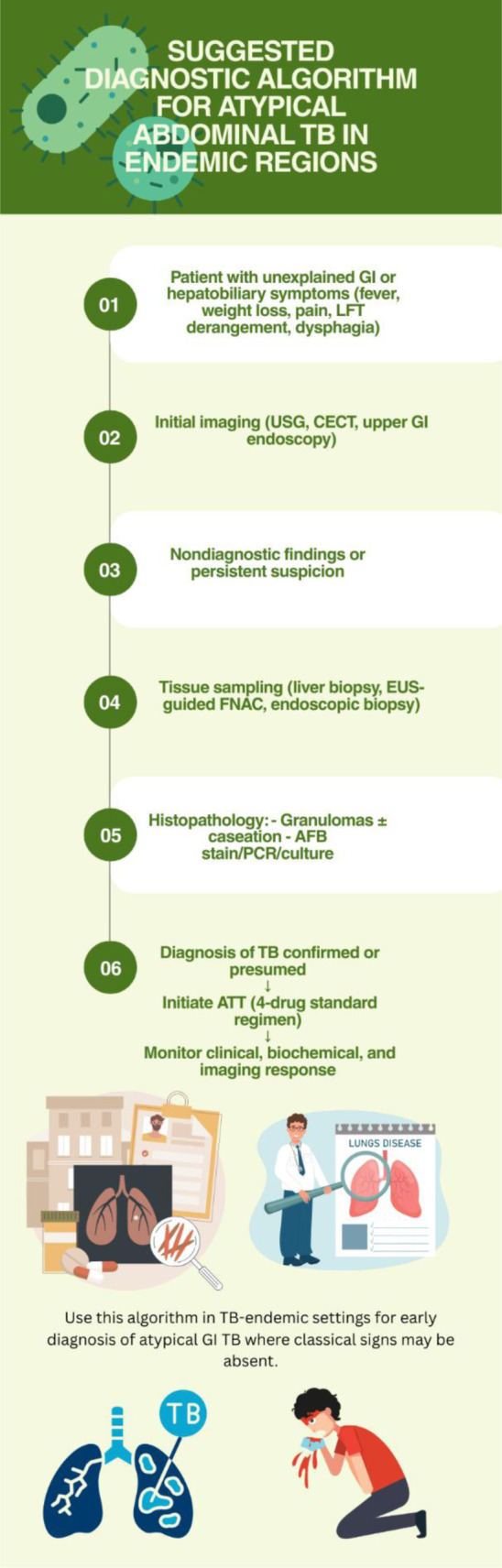
Flowchart of suggested diagnostic algorithm for atypical abdominal TB in endemic regions

Diagnostic algorithm for suspected atypical GI TB (Figure 5(Fig. 5))

## Declaration

### Statements & declarations

The authors declare that no funds, grants, or other support were received during the preparation of this manuscript.

### Funding

The authors did not receive support from any organization for the submitted work.

### Financial interests

The authors declare they have no financial interests.

### Artificial Intelligence (AI) - ) - assisted technology

AI tools were used solely for language refinement and stylistic editing, without any role in data generation, analysis, interpretation, or figure creation.

### Author contributions

**AY, YS: **Conceptualization, supervision, validation, writing-original draft, visualization, project administration, writing - review & editing.

## Supplementary Material

Supplementary information

## Figures and Tables

**Table 1:**
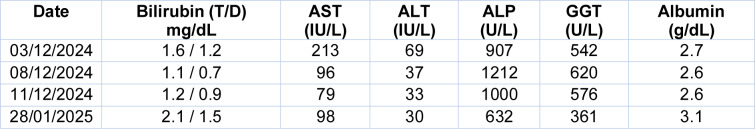
Timeline of liver function parameters pre-treatment

**Table 2:**
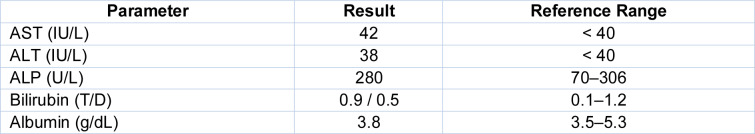
Follow-up liver function at 8 weeks post-ATT

**Table 3:**
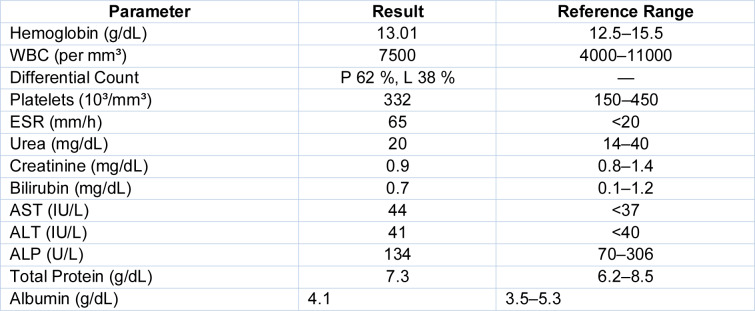
Baseline blood investigations

**Table 4:**
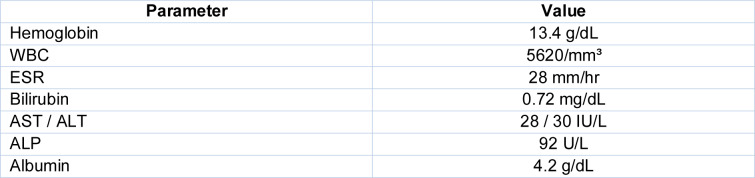
Follow-up laboratory results at 8 weeks

**Table 5:**
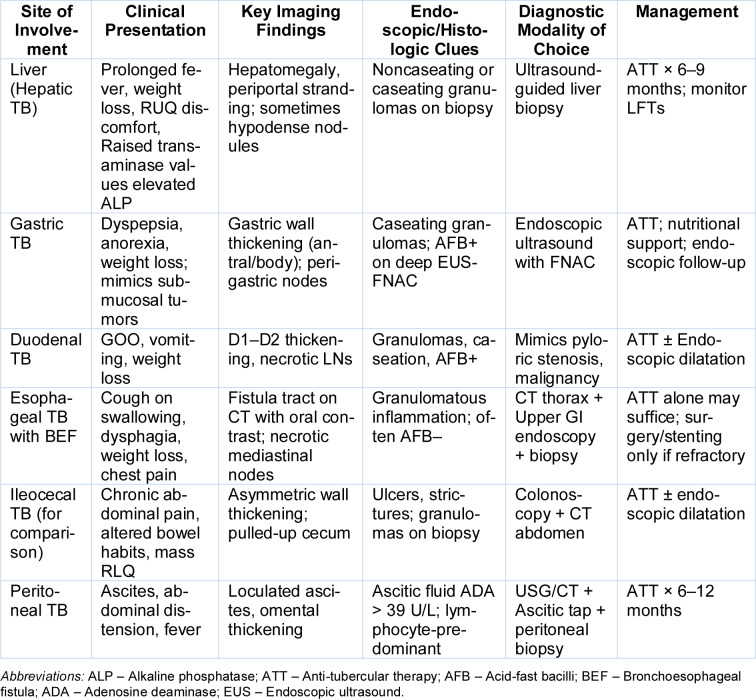
Rare gastrointestinal TB manifestations: diagnostic clues and management principles

**Table 6:**
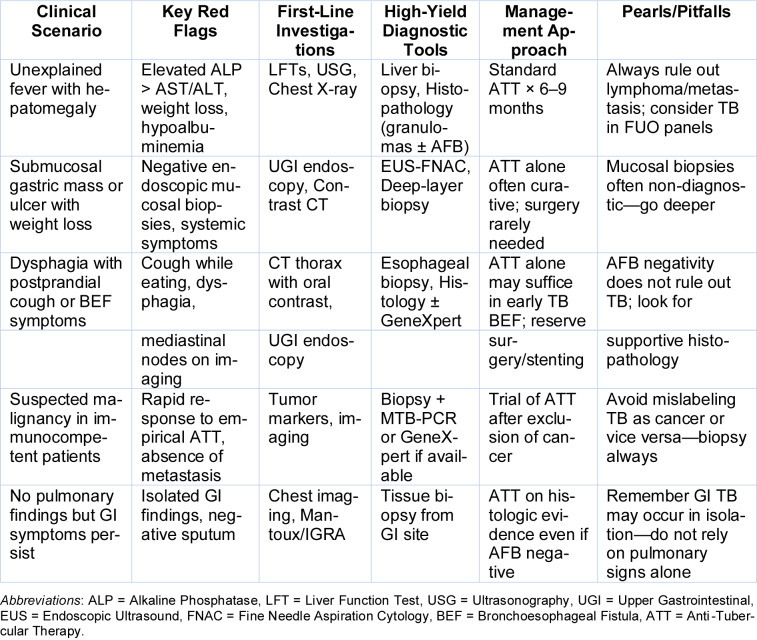
Clinical clues and diagnostic strategy for atypical gastrointestinal tuberculosis in endemic areas

**Table 7:**
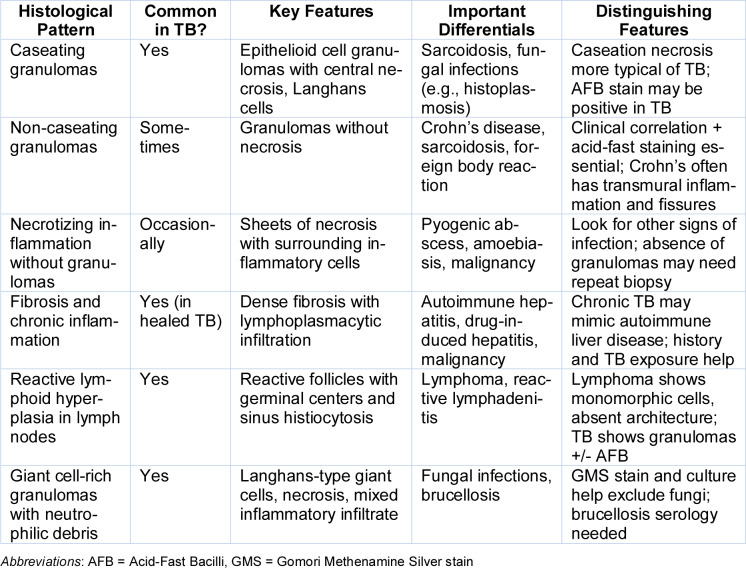
Histopathological features and differential diagnosis in abdominal tuberculosis
