# Supplementary material for: The hidden path of mycobacterium: a case series of rare and elusive manifestations of gastrointestinal tuberculosis
Source: EXCLI J. 2026 Jan 5;25:35–49. doi: 10.17179/excli2025-9040 (PMC12901959; doi:10.17179/excli2025-9040)
Supplement: Supplementary information [file EXCLI-25-35-s-001.pdf]

EXCLI Journal 2026;25:35-49 – ISSN 1611-2156
Received: October 21, 2025, accepted: November 15, 2025, published: January 05, 2026

Supplementary information to:

Case report:

THE HIDDEN PATH OF MYCOBACTERIUM: A CASE SERIES OF RARE AND ELUSIVE MANIFESTATIONS OF GASTROINTESTINAL TUBERCULOSIS

Nitika Yadav[1], Abhishek Yadav[1], Neharica Joshi[1], Shubhangi Gupta[1], Yashendra Sethi[1,2,3,*]

[1] Subharti Medical College, Swami Vivekanand Subharti University, Meerut, India
[2] PearResearch, Dehradun, India
[3] Lumen Foundation, Florida, US

* **Corresponding author:** Dr. Yashendra Sethi, Subharti Medical College, Swami Vivekanand Subharti University, Meerut, India. E-mail: yash@pearresearch.com

https://dx.doi.org/10.17179/excli2025-9040

This is an Open Access article distributed under the terms of the Creative Commons Attribution License (https://creativecommons.org/licenses/by/4.0/).

EXCLI Journal 2026;25:35-49 – ISSN 1611-2156
Received: October 21, 2025, accepted: November 15, 2025, published: January 05, 2026

Supplementary Table 1: Summary of reported cases of duodenal tuberculosis with gastric outlet obstruction

Authors, year	Country of author	Age/sex	Symptoms	Onset time	PTB	Part of lesion	Pre-treatment diagnosis	Treatment (surgical procedure)	Diagnostic method (tool of Bx)	Response to treatment
Ahmad and Ahmed, 1985	Pakistan	22/M	Bowel obstruction, Fever	4 M	(−)	D3	–	Operation → ATT (Duodenojejunostomy)	Pathological (Surgical)	–
		8/M	Vomit, Abdominal pain	Few M	(−)	D3	–	ATT only	Empirical treatment	1 M for weight gain
Kriplani et al., 1986	India	45/M	Fullness, Vomit, Weight loss	6 M	(+)	D3	–	Operation → ATT (Gastroenterostomy, Truncal vagotomy)	Pathological (Surgical)	–
Mani and Rananavare, 1996	India	18/M	Epigastric pain, Vomit	6 M	(+)	D2	–	Operation → ATT (Gastroenterostomy, Truncal vagotomy)	Pathological (Surgical)	6 M for symptom free
Rautou et al., 2005	French	32/M	Abdominal pain, Esophagitis, Duodenal stenosis	–	(−)	D2	Zollinger–Ellison syndrome	Operation → ATT (Pancreatoduodenectomy)	Pathological (Surgical)	–
Benzekri et al., 2008	Morocco	60/M	Abdominal pain, Vomit, Weight loss	1 M	(−)	D1	–	Operation → ATT (Gastroenterostomy)	Pathological (Surgical)	11 M for symptom improvement
		57/M	Epigastric pain, Vomit, Weight loss	6 M	(−)	D3	–	Operation → ATT (Duodenojejunectomy, Duodenojejunostomy)	Pathological (Surgical)	3Y for symptom free

EXCLI Journal 2026;25:35-49 – ISSN 1611-2156
Received: October 21, 2025, accepted: November 15, 2025, published: January 05, 2026

Flores et al., 2011	Philippines	31/M	Epigastric pain, Vomit	1Y	(−)	D1	Peptic ulcer	Operation → ATT (Gastrojejunostomy)	Pathological (Surgical)	3 M for symptom free
Al-Hilou et al., 2011	UK	62/F	Heartburn, Chest discomfort, Malaise, Weight loss	9 M	(−)	D2	Suspicious of TB	ATT only	Pathological, Bacteriological (EUS-FNA, Surgical)	–
Chawla et al., 2012	India	42/M	Epigastric pain, Fullness, Vomit	25D	(−)	D3	–	Operation → ATT (Gastrojejunostomy, Jejunojejunostomy)	Pathological (Surgical)	–
Padmanabhan et al., 2013	UK	33/M	Dyspepsia, GOO, Weight loss	3Y	(−)	D1, D2	Crohn's disease or TB or Peptic ulcer	Endoscopic balloon dilation + ATT	Bacteriological (Endoscopic)	3 M for improvement of endoscopic findings
Sisodiya and Ramachandra, 2013	India	35/F	Fullness, Vomit, Weight loss	3Y	(−)	D3	SMA syndrome, Duodenal stricture	Operation → ATT (Jejunum resection, Duodenojejunostomy, Ileocecal resection)	Pathological (Surgical)	–
Fatemi et al., 2014	Iran	18/F	Abdominal pain, Nausea, Vomit, Fatigue, Powerless, Weight loss	4 M	(−)	D2, D3	Retroperitoneal lymphoma or GIST or Desmoid tumor	Operation → ATT (Gastrojejunostomy)	Pathological, Bacteriological, PCR (Surgical)	–

EXCLI Journal 2026;25:35-49 – ISSN 1611-2156
Received: October 21, 2025, accepted: November 15, 2025, published: January 05, 2026

Kalpande et al., 2017	India	13/M	Vomit, Fever, Epigastric pain, Weight loss	14D	(−)	D1, D2	Peptic ulcer	Operation → ATT (Gastrojejunostomy)	Pathological (Surgical)	3 M for partial improvement of endoscopic findings
Lee et al., 2017	Korea	47/F	Vomit, Weight loss	1 M	(+)	D3, D4	TB	ATT → Operation (Laparoscopic duodenojejunostomy)	PCR (Endoscopic)	–
Udgirkar et al., 2018	India	24/F	Vomit, Fever, Weight loss	1 M	(−)	D1, D2	TB	ATT only	Pathological, Bacteriological (Endoscopic)	6 M for improvement of endoscopic findings
		22/F	Upper abdominal pain, Vomit	1 M	(−)	D1	TB	ATT only	Bacteriological (Endoscopic)	5 M for improvement of endoscopic findings
Meregildo-Rodríguez et al., 2019	Peru	31/M	Fullness, Epigastric pain, Nausea, Vomit, Weight loss	4 M	(−)	D2, D3	Duodenal stricture	ATT only	Pathological (Endoscopic)	2 M for symptom improvement
Chang et al., 2020	Thailand	52/M	Abdominal pain, Early satiety, Weight loss	3 M	(−)	D2	Peptic stricture	Endoscopic balloon dilation → ATT	Bacteriological (Endoscopic)	6 M for some improvement and 12 M for complete resolution of endoscopic findings

EXCLI Journal 2026;25:35-49 – ISSN 1611-2156
Received: October 21, 2025, accepted: November 15, 2025, published: January 05, 2026

Molla et al., 2023	Ethiopia	48/M	Vomit, Epigastric pain, Weight loss	1Y	(−)	D2, D3	TB	ATT only	Pathological (Endoscopic)	6 M for symptom improvement
Sato et al., 2024	Japan	35/M	Epigastric pain, Fullness, Vomit, Weight loss	1 M	(+)	D1, D2	TB	Operation → ATT (Laparoscopic gastrojejunostomy)	Pathological (EUS-FNA), Bacteriological (Septum PCR)	Persistent stenosis at 6 M endoscopy
Our case (Yadav et al., 2026; this publication)	India	24?M	Vomiting, Pain abdomen, Fullness, Weight loss	1M	(+)	D1-d2 junction	TB	ATT	Pathological (Endoscopic)	6 M for complete symptomatic resolution

Abbreviations: ATT Antituberculosis treatment, *Bx* biopsy, *D* Days, *EUS-FNA* Endoscopic ultrasound-guided fine-needle aspiration, *GIST* Gastrointestinal stromal tumor, *GOO* Gastric outlet obstruction, *M* Months, *PCR* Polymerase chain reaction, *PTB* Pulmonary tuberculosis, *SMA* Superior mesenteric artery, *TB* Tuberculosis, *UK* United kingdom, *Y* Years, – Not mentioned

REFERENCES

Ahmad M, Ahmed M. Duodenal tuberculosis. J Pak Med Assoc. 1985;35(2):53–54.

Al-Hilou H, Carroll N, Jah A, Davies S, Parkes M. A rare cause of duodenal stricture. BMJ Case Rep. 2011; 2011:bcr1020103379. doi: 10.1136/bcr.10.2010.3379.

Benzekri O, Mouhadi SE, Chourak M, Boussetta S, Absi ME, Echarab M, et al. Tuberculous duodenal stenosis: report of two cases. Case Rep Gastroenterol. 2008;2(3):444-50. doi: 10.1159/000161566.

Chang A, Chantarojanasiri T, Pausawasdi N. Duodenal tuberculosis: uncommon cause of gastric outlet obstruction. Clin J Gastroenterol. 2020;13:198–202. doi: 10.1007/s12328-019-01007-4.

Chawla I, Aery V, Singh K, Singla B, Singh V. Duodenal tuberculosis presenting as gastric outlet obstruction. J Surg Case Rep. 2012;2012(7):13. doi: 10.1093/jscr/2012.7.13.

Fatemi SR, Ghobakhlou M, Alizadeh L. Obstructive pseudotumor of tuberculosis in a young woman: a rare presentation. Case Rep Gastrointest Med. 2014;2014: 914253. doi: 10.1155/2014/914253.

Flores HB, Zano F, Ang EL, Estanislao N. Duodenal tuberculosis presenting as gastric outlet obstruction: a case report. World J Gastrointest Endosc. 2011;3(1): 16–19. doi: 10.4253/wjge.v3.i1.16.

Kalpande S, Pandya JS, Tiwari A, Adhikari D. Gastric outlet obstruction: an unusual case of primary duodenal tuberculosis. BMJ Case Rep. 2017;2017: bcr2016217966. doi: 10.1136/bcr-2016-217966.

Kriplani AK, Kumar S, Sharma LK. Obstruction of the third part of the duodenum in tuberculosis. Postgrad Med J. 1986;62(731):879–880. doi: 10.1136/pgmj.62.731.879.

Lee JM, Kim ES, Chun HJ. Gastrointestinal: unexpected cause of refractory vomiting. J Gastroenterol Hepatol. 2017;32(3):543. doi: 10.1111/jgh.13584.

Mani S, Rananavare R. Duodenal tuberculosis. Eur J Radiol. 1996;23(2):102–103. doi: 10.1016/0720-048X(96)01041-8.

Meregildo-Rodríguez E, Méndez-Florián K, Espino-Saavedra W. Duodenal obstruction and upper gastrointestinal bleeding as the initial presentation of an isolated duodenal tuberculosis. Rev Chilena Infectol. 2019;36(3):387–391. doi: 10.4067/S0716-10182019000300387.

Molla YD, Kassa SA, Tadesse AK. Rare case of duodenal tuberculosis causing gastric outlet obstruction: a case report. Int J Surg Case Rep. 2023;105:108080. doi: 10.1016/j.ijscr.2023.108080.

Padmanabhan H, Rothnie A, Singh P. An unusual case of gastric outlet obstruction caused by tuberculosis: challenges in diagnosis and treatment. BMJ Case Rep. 2013;2013:bcr2012008277. doi: 10.1136/bcr-2012-008277.

Rautou PE, Corcos O, Hammel P, Cazals-Hatem D, Slama JL, Morin AS, et al. Pseudo-syndrome de Zollinger-Ellison en rapport avec une sténose duodénale d'origine tuberculeuse [Pseudo Zollinger-Ellison syndrome in a patient with duodenal stenosis caused by tuberculosis]. Gastroenterol Clin Biol. 2005; 29(11):1164-8. French. doi: 10.1016/s0399-8320(05)82183-9.

Sato N, Shiobara M, Wakatsuki K, Suda K, Miyazawa K, Aida T, et al. Duodenal tuberculosis with gastric outlet obstruction: a case report of successful diagnosis and treatment, with review of literature. Surg Case Rep. 2024;10(1):42. doi: 10.1186/s40792-024-01840-x.

Sisodiya R, Ramachandra L. Tubercular duodenal, jejunal and ileocecal stricture in a patient. BMJ Case Rep. 2013;2013:bcr2013200347. doi: 10.1136/bcr-2013-200347.

Udgirkar S, Surude R, Zanwar V, Chandnani S, Contractor Q, Rathi P. Gastroduodenal Tuberculosis: A Case Series and Review of Literature. Clin Med Insights Gastroenterol. 2018;11:1179552218790566. doi: 10.1177/1179552218790566.
